# Treatment of tibial traumatic osteomyelitis with negative pressure closure drainage combined with open bone grafting or bone migration and its effect on the levels of CRP, TNF-α and IL-6 in the serum

**DOI:** 10.4314/ahs.v23i3.55

**Published:** 2023-09

**Authors:** Xinqiang Wang, Liangbang Wu, Yuehong Zhang, Zhenhai Hou, Longbao Zheng, Zenghui Gu

**Affiliations:** Department of third Orthopaedics, The 903 Hospital of the Chinese people's Liberation Army, Hangzhou,310004, China

**Keywords:** Bone removal, CRP, IL-6, Negative pressure closure drainage, Tibia, TNF-α, Traumatic osteomyelitis

## Abstract

**Objective:**

To observe the effect of negative pressure closure drainage combined with open bone grafting or bone migration in the treatment of tibial traumatic osteomyelitis.

**Methods:**

Eighty-six (86) cases of tibial traumatic osteomyelitis admitted to Hospital of the Chinese people's Liberation Army from September 2017 to September 2018 were randomly divided into control group and observation group, forty-three (43) cases each. Debridement, open bone grafting or bone migration was given to the control group.

The observation group was treated with negative pressure closed drainage on the basis of the control group. In addition, the serum components such as CRP, TNF-α, IL-6 of the control group and observation group were detected respectively after surgery.

**Results:**

Observation group granulation cover wound, fracture healing time was shorter than the control group, fracture healing rate was better than the control group, the difference has statistical significance (P<0.05). By comparing serum CRP, TNF-α, IL-6 levels before and after treatment in the two groups, it was found that the observation group was lower than the control group (P<0.05, respectively). Serum CRP, TNF-α, IL-6 levels were significantly (P<0.05).

**Conclusion:**

The treatment of tibial traumatic osteomyelitis with negative pressure closure drainage combined with open bone grafting or bone displacement has a good effect on fracture healing and is worth popularizing.

## Introduction

With the progress of society and the development of economy, tibial fractures caused by high-energy injuries are increasing day by day, among which, open fractures are more common 1,2.

If the open tibial fracture is treated inappropriately in the early stage, it is easy to cause local soft tissue suppurative infection, ischemic necrosis at the fracture end, bone non-union and sinus tract formation, and then develop into traumatic osteomyelitis. Such patients often have extensive scar adhesion or severe soft tissue defects. At present, the commonly used treatment methods are antibiotics and surgical debridement to control the active infection, then local flap transfer or free flap transplantation to close the wound surface and finally bone grafting or bone relocation to repair the bone defect. However, this method has some shortcomings, such as long treatment cycle, many surgeries, high cost and large trauma 3,4.

About 1/3 of the tibia is subcutaneous and lacks muscle tissue coverage which is prone to fracture. Moreover, the blood supply at both ends of the tibia is relatively rich. It is likely to cause local soft tissue supportive infection if not handled in the earl stage and the fracture end is ischemic necrosis, which then develops into traumatic osteomyelitis.

Due to traumatic osteomyelitis, both tibial fracture, infection causing fracture healing, and fractures caused by soft tissue defect, scar adhesion, bone exposed, and the internal fixation of fracture fixation, infection control, increasing infection, forming a vicious cycle, leading to disease treatment duration are long, difficult, and have poor effects 5,6. The purpose of this study was to observe the effects of negative pressure closure drainage combined with open bone grafting or bone removal in patients with tibial traumatic osteomyelitis.

## Materials and methods

### General information

There were 86 cases of tibial traumatic osteomyelitis patients admitted to Hospital of the Chinese people's Liberation Army from September 2017 to September 2018 were selected as the study objects which all met the clinical diagnostic criteria for traumatic osteomyelitis. There was no surgical contravention, coagulation dysfunction, diabetes mellitus, local bone exposure, skin sinus tract, and soft tissue injuries. The patients were randomly divided into control group and observation group, 43 cases each. There were 25 males and 18 females in the control group. The average age was 46.1±13.8 years. In the observation group, there were 19 females and 24 males, aged from 26 to 70 years, with an average age of 49.3±12.7 years. There was no statistically significant difference between the two groups in general data (P > 0.05), suggesting comparability.

### Treatment

The control group after admission to clean up the wound and anti-infection therapy with antibiotics in patients with wound granulation tissue cover and no solutions, in patients with bone defect is less than 4cm below the line open for the treatment of bone graft, in patients with autogenous iliac cancellous bone, rinsed repeatedly with hydrogen peroxide and sodium chloride solution, cut into the appropriate size of bone, filling bone defect area. Bone removal was performed for bone defects over 4 cm. External fixation stent was installed after debridement, proximal and distal tibial osteotomy was performed according to the defect site. Fibula was amputated at the corresponding site for limb extension. X-ray examination was performed every 2 weeks to observe the fracture healing. In the observation group, the injured limb was bandaged, the balloon tourniquet was used for hemostasis, the original internal fixation was removed, the necrotic tissue on the wound surface was cleared, the pulp cavity was opened, the necrosis and hardened bone were removed, the tourniquet was released and debridement continued until fresh blood exudated from the bone end and the wound margin. The external fixation stent was fixed and the negative pressure sealing drainage material was used to cover the wound according to the size of the wound. The wound was closed with a biological permeable membrane and the negative pressure was connected to the center. After the granulation tissue was formed on the wound surface, the same open bone grafting or bone migration as the control group was performed. After the operation, continuous negative pressure closure drainage was performed, excipients were replaced once a week and X-ray examination was performed once every 2 weeks to observe the fracture healing.

### Observation indicators

The fracture healing of the two groups was evaluated by comparing the time spent in fracture healing and granulation covering the wound surface. According to Paley's score for fracture healing, the results were classified as excellent, good, moderate and poor.

### Detection of serum CRP, TNF-α, IL-6

Three days after treatment, the patient's blood was collected and serum levels of CRP, TNF-α and, IL-6 were detected.

### Follow-up

Follow-up X-ray film was taken 1month, 2months, 5months, 1year or more after the operation. The follow-up focused on the X-ray healing status and the verified control status, including the sensory perception, mechanical movement and function of the affected limb.

### Statistical analysis

SPSS18.0 statistical software was used for data processing and analysis. The quantitative data were expressed as (X±S). t-test was used for inter-group comparison and 2 test was used for inter-group comparison of qualitative data. P < 0.05 was considered statistically significant.

## Results

### Healing time

Granulation coverage of the wound and fracture healing time in the observation group were shorter as compared to control group with statistically significant differences (P < 0.05) (Table 1).

**Table 1:** comparison of healing time between the two groups (X±s)

Group	n	Granulation covering wound time/d	Fracture healing time/month
Control group	43	47.2±3.5	9.2±1.0
Observation group	43	30.7±2.8	6.3±0.5

### Healing effect

The rate of excellent and good fracture healing in the observation group was higher as compared to control group, and the difference was statistically significant (P < 0.05) (Table 2).

**Table 2:** Comparison of fracture healing effect between the two groups (n, %)

Group	n	Excel lent	Good	Average	Poor	Ratio of excellent and good
Control group	43	30	10	3	0	95.3
Observation group	43	24	11	7	1	83.7

### Follow-up and prognosis

Eighty-six patients were followed up, with an average follow-up of 16.1 months (7-23), of whom, 10 patients presented axial tibial deviation after surgery, and timely correction were recovered.

In 15 patients, the skin was pulled by the steel needle during the operation and the pain was felt. In this case, the bone was stopped for 3-5 days and the bone was prolonged after the pain. There were no cases of vascular nerve damage. All the patients were eventually cured of traumatic osteomyelitis and all the patients had good therapeutic effect.

### Effects on serum levels of CRP, TNF-α, IL-6

After treatment, the serum levels of CRP, TNF-α and IL-6 were compared in the two groups. It was found out that the observation group was lower than the control group (P<0.05, respectively). Serum CRP, TNF-α and IL-6 were significantly reduced after treatment (P<0.05).

## Discussion

There are both fractures and infections in tibial traumatic osteomyelitis, the treatment of the disease focuses on the elimination of dead space, the removal of lesions, and the promotion of bone healing 7.

In the treatment, antibiotics and surgical debridement are used in the first place to control the infection. However, in recent years it has been found that closing the wound surface with skin flap not only has the possibility of skin flap necrosis but also causes iatrogenic injury to the normal part of the patient, resulting into the loss of tissue and function of the donor area of the flap 8. Therefore, the present clinical advocates simplified wound treatment. After the removal of necrotic tissue, antibiotics were used to fight infection and the wound surface was covered with granulation tissue instead of the flap to close the wound surface 9-13. Some findings have proposed the use of negative pressure closure attraction in treatment, which can promote the granulation tissue covering the wound surface, reduce the occurrence of infection, and promote fracture healing 14.

The results of the present study showed that the granulation coverage of the wound in the observation group. The fracture healing time was shorter than that in the control group, and the excellent and good rate of fracture healing was higher than that in the control group, with statistically significant differences (P<0.05). The results showed that negative pressure closure and suction therapy could promote fracture healing and reduce the incidence of infection. Current clinical bone graft with open bone graft, bone removal technique, which is much used for bone defect more than 4 cm, its small trauma, less pain, less bleeding, can be a one-time bone and soft tissue repair, shortening the time of trauma care, good for patients of soft tissue, fracture healing, but shortcomings for the treatment time is long, external fixation support to adjust. Open bone grafting does not close the wound surface after debridement, and after the wound surface is covered with fresh granulation tissue, the spongiform bone of the iliac crest is taken to repair the bone defect. This treatment can effectively avoid complex soft tissue reconstruction, but the disadvantage is that it may cause the patient to become more infected and the bone grafting fails.

Some findings have proposed that the combination of negative pressure closure and attraction during treatment can completely seal the wound surface and avoid cross-infection. At the same time, continuous attraction can remove necrotic tissue and exudate which is conducive to the recovery of wound microcirculation, thereby promoting the growth of granulation tissue, promoting angiogenesis and accelerating wound healing. To attract negative pressure closed and open joint bone graft can effectively avoid infection, reduce the switching frequency, thus reducing the article and bone necrosis, loss and removal of bone joint can not only repair bone defect but also keep body length, fracture fixation and to avoid the repeated infection caused by the long-time treatment, can accelerate fracture healing, shorten the treatment cycle. In addition, the results of this study showed that the serum levels of CRP, TNF-α, IL-6 levels in the observation group were all lower than those in the control group after treatment, suggesting that this treatment method was related to the higher secretion of the three factors.

In conclusion, the treatment of tibial traumatic osteomyelitis with negative pressure closure drainage combined with open bone grafting or bone migration has a good fracture healing effect in patients which is worthy of popularization and application.

## Figures and Tables

**Table 3:** comparison of serum CRP, TNF-α, IL-6 levels between the two groups after treatment

Group	Control	Observation	X^2^	P
CRP (mg/L)	10.23±3.44	5.43±1.12	3.8	0.002
TNF-α (ng/L)	13.68±3.23	9.50±2.44	4.9	0.031
IL-6 (ng/L)	9.23±2.36	6.35±1.38	6.9	0.001

Note: P<0.05 indicate significant difference
